# Impact of Germline *BRCA1/2* Mutations on Clinical Outcomes in Metastatic Triple-Negative Breast Cancer Patients Treated with Sacituzumab Govitecan—An International CEBCC Study from Poland, the Czech Republic and Slovakia

**DOI:** 10.32604/or.2026.076384

**Published:** 2026-05-21

**Authors:** Renata Pacholczak-Madej, Mirosława Püsküllüoğlu, Anna Polakiewicz-Gilowska, Małgorzata Pieniążek, Iveta Kolářová, Miroslava Malejčíková, Lenka Rušinová, Miloš Holánek, Renata Soumarová, Karolina Winsko-Szczęsnowicz, Justyna Żubrowska, Aleksandra Konieczna, Agnieszka Młodzińska, Daniel Krejčí, Iwona Danielewicz, Magdalena Szymanik-Resko, Tomasz Ciszewski, Maja Lisik-Habib, Anika Pękala, Hana Študentová, Jan Šustr, Bogumiła Czartoryska-Arłukowicz, Aleksandra Łacko, Jolanta Smok-Kalwat, Michał Jarząb, Zuzana Bielčiková, Marcin Kubeczko

**Affiliations:** 1 Department of Gynecological Oncology, Maria Sklodowska-Curie National Research Institute of Oncology, Kraków Branch, Kraków, Poland; 2 Department of Anatomy, Jagiellonian University Medical College, Krakow, Poland; 3 Department of Clinical Oncology, Maria Sklodowska-Curie National Research Institute of Oncology, Kraków Branch, Kraków, Poland; 4 Breast Cancer Center, Maria Sklodowska-Curie National Research Institute of Oncology, Gliwice, Poland; 5 Lower Silesian Comprehensive Cancer Center, Wrocław Medical University, Wrocław, Poland; 6 Department of Oncology and Radiotherapy, Faculty of Medicine in Hradec Králové and University Hospital in Hradec Králové, Charles University, Hradec Králové, Czech Republic; 7 Oncology Clinic of LFUK, National Cancer Institute, Bratislava, Slovakia; 8 Department of Oncology, Stefan Kukura Hospital Michalovce, Michalovce, Slovakia; 9 Department of Comprehensive Cancer Care, Faculty of Medicine, Masaryk University, and Masaryk Memorial Cancer Institute, Brno, Czech Republic; 10 Department of Oncology, Third Faculty of Medicine, Charles University, University Hospital Kralovské Vinohrady, Prague, Czech Republic; 11 Department of Clinical Oncology, Maria Sklodowska-Curie Bialystok Oncology Center, Białystok, Poland; 12 Department of Clinical Oncology, Holy Cross Cancer Center, Kielce, Poland; 13 Department of Breast Cancer and Reconstructive Surgery, Maria Sklodowska-Curie National Research Institute of Oncology, Warsaw, Poland; 14 Department of Oncology, First Faculty of Medicine, Charles University in Prague, and Bulovka University Hospital, Prague, Czech Republic; 15 Department of Oncology and Radiotherapy, Szpitale Pomorskie sp. z o.o., Gdynia, Poland; 16 Department of Metabolic Diseases and Immuno-Oncology, Medical University of Lublin, Lublin, Poland; 17 Department of Proliferative Diseases, Nicolaus Copernicus Multidisciplinary Centre for Oncology and Traumatology, Lodz, Poland; 18 Department of Oncology, Faculty of Medicine and Dentistry, Palacky University and University Hospital, Olomouc, Czech Republic; 19 Department of Oncology and Radiotherapy, Faculty of Medicine in Pilsen, Charles University, and University Hospital Pilsen, Plzen, Czech Republic; 20 Department of Oncology, First Faculty of Medicine, Charles University, and General University Hospital, Prague, Czech Republic

**Keywords:** Triple-negative breast cancer, sacituzumab govitecan, *BRCA1/2* germline mutations, real-world evidence, international multicenter cohort

## Abstract

**Background:** Germline breast cancer susceptibility gene 1/2 (*BRCA1/2*) variants guide breast cancer treatment, but their clinical relevance in metastatic triple-negative breast cancer (mTNBC) treated with sacituzumab govitecan (SG) remains unclear. The study aimed to evaluate the association between *BRCA* status and outcomes in SG-treated mTNBC. **Methods:** We retrospectively analyzed 264 patients with mTNBC and known germline *BRCA1/2* (*gBRCA1/2*) status who received SG between August 2021 and May 2025 across multiple oncology centers in Poland, the Czech Republic and Slovakia. Survival outcomes were compared between patients with g*BRCA1/2* mutations (*gBRCA1/2m*) and those with g*BRCA1/2* wild-type (g*BRCA1/2*wt) using Kaplan–Meier estimates, the log-rank test, and multivariable Cox proportional hazards models. Two-sided *p* < 0.05 was considered statistically significant. **Results:** Among 264 patients, 35 (13.3%) were g*BRCA1/2*m and 229 (86.7%) were g*BRCA1/2*wt. After a median follow-up of 9.9 months, the median progression-free survival (PFS) was 4.5 months (95% confidence interval [CI] 2.1–6.3) in g*BRCA1/2* carriers versus 4.2 months (95% CI 3.5–5.8) in g*BRCA1/2*wt patients (*p* = 0.10). Median overall survival (OS) was 9.1 months (95% CI 5.0–15.1) in g*BRCA1/2* carriers compared to 11.5 months (95% CI 10.3–13.5) in g*BRCA1/2*wt patients (*p* = 0.26). Brain metastases were more frequent in carriers (20% vs. 8.3%, *p* = 0.06). In multivariable analysis, Eastern Cooperative Oncology Group (ECOG) performance status was the only independent predictor of poorer survival (hazard ratio 1.97, 95% CI 1.42–2.74, *p* < 0.01), while g*BRCA1/2* status showed no independent association. **Conclusions:** In this large retrospective cohort of mTNBC patients treated with SG, the presence of g*BRCA1/2* was not associated with statistically significant differences in PFS or OS.

## 1 Introduction

Triple-negative breast cancer (TNBC) is the most aggressive breast cancer subtype, accounting for 10–15% of all cases [1]. It is more common in younger women and is frequently associated with germline breast cancer susceptibility gene 1 (*BRCA1*) or *BRCA2* mutations (g*BRCA1/2*m) [2,3]. The absence of hormone receptors (HR) and negative human epidermal growth factor receptor 2 (HER2) status underlines the therapeutic challenges in this disease. Historically, chemotherapy was the only option in both curative and palliative settings; however, the advent of antibody–drug conjugates (ADCs) and immunotherapy has changed current management [1,4,5].

Mutations in the *BRCA1* and *BRCA2* tumor suppressor genes account for the majority of hereditary breast cancers. The lifetime risk of developing breast cancer by age 70 is 55–72% for individuals with *BRCA1*m tumors and 45–69% for individuals with *BRCA2*m tumors, compared with ~12% in the general population [3]. The histopathologic features of *BRCA1*m tumors are well characterized: they often display medullary features, are of higher histologic grade, and commonly present as TNBC, with brain metastases occurring more frequently [2,3]. Clinically, these tumors are more sensitive to poly(ADP-ribose) polymerase (PARP) inhibitors (PARPi) and DNA-damaging agents such as platinum-based chemotherapy, while showing lower responsiveness to cyclin-dependent kinase 4/6 (CDK4/6) inhibitors [6,7].

Across unselected breast cancer populations, g*BRCA1/2* status has not consistently emerged as a prognostic factor for overall survival (OS) [8]. Nevertheless, g*BRCA1/2*m have been associated with poorer prognosis in HER2-positive and in HR-positive/HER2-negative disease [9,10], whereas a survival advantage has been suggested in TNBC with g*BRCA1/2*m [10]. Pathogenic g*BRCA1/2*m are associated with higher risks of recurrence and death in breast cancer. Notably, outcomes also differ between carriers: *BRCA2*m generally have more favorable invasive disease-free survival and OS than *BRCA1*m, consistent with earlier onset and a predominant triple-negative phenotype in *BRCA1*m versus older age and predominantly hormone receptor–positive disease in *BRCA2*m [11].

Sacituzumab govitecan (SG) is an ADC comprising an IgG1κ antibody to trophoblast cell-surface antigen 2 (TROP-2) linked to the topoisomerase I inhibitor [4,5]. SG is recommended by international guidelines [12,13] for metastatic or locally advanced TNBC after ≥2 prior systemic regimens based on the ASCENT trial, which showed OS and progression-free survival (PFS) benefit over the physician’s choice chemotherapy [14]. The efficacy of SG is also supported by different real-world data [15,16,17]. A second approval is for heavily pretreated, endocrine-resistant, HR-positive, HER2-negative metastatic or inoperable breast cancer, based on the results of the TROPiCS-02 trial [18].

SN-38, the active metabolite of irinotecan and the payload of SG, traps topoisomerase I–DNA complexes and induces replication-associated double-strand breaks. Tumors with defective homologous recombination (“BRCAness”) are more susceptible to topoisomerase I inhibition, suggesting that DNA repair and checkpoint context—rather than germline *BRCA* status alone—may shape therapeutic response [19]. Preclinical TNBC models further show that SG combined with PARPi produces greater DNA damage and growth arrest than either agent alone, with benefits observed irrespective of *BRCA1/2* status and acceptable tolerability. These data provide a biological rationale to examine SG outcomes across *BRCA* subgroups in metastatic TNBC [20].

The prognostic impact of g*BRCA1/2* mutation status in metastatic TNBC treated with SG remains unknown. This study aims to address this gap by examining outcomes in patients from Eastern Europe (Poland, the Czech Republic and Slovakia) harboring g*BRCA1/2* mutations and treated with SG.

## 2 Methods

### 2.1 Study Design and Setting

We performed a retrospective, multicenter cohort analysis under the CEBCC-102 initiative (Central European Breast Cancer Collaboration), a Central European real-world program collecting outcomes of sacituzumab govitecan (SG) in metastatic triple-negative breast cancer (mTNBC) and previously used for biomarker-focused evaluations in this setting [21]. The study included 264 women with mTNBC treated with SG in the second or later palliative line between August 2021 and May 2025; the data cut-off was 20 June 2025. Patients were contributed by 18 oncology centers in Poland, the Czech Republic and Slovakia. SG eligibility in routine practice followed key principles of the ASCENT trial and was broadly comparable to its main criteria [14]. Reporting followed the Strengthening the Reporting of Observational Studies in Epidemiology (STROBE) guidance for cohort studies; the checklist is provided in the Supplementary Materials [22].

### 2.2 Ethical Considerations

The study protocol received approval from the relevant research ethics committees: Maria Skłodowska-Curie National Research Institute of Oncology, Kraków (protocol 2/2023, 18 April 2023) and Warsaw (protocol 21/2024, 22 February 2024); Masaryk Memorial Cancer Institute, Brno (approval 1737/2025, 10 June 2025); the Ethics Committee of the General University Hospital in Prague (protocol 110825 S-IV, 21 August 2025); the Ethics Committee of National Cancer Institute, Bratislava, Slovakia (22 September 2025). A waiver of study-specific informed consent was granted by the relevant Bioethics Committees because of the retrospective nature of the study and the use of anonymized data. All patients had previously provided institutional consent for SG treatment and for the use of clinical data in academic research, according to local regulations.

### 2.3 Eligibility Criteria

Patients were eligible for this study if they met all the following: histologically confirmed TNBC defined as estrogen receptor (ER) < 1%, progesterone receptor (PR) < 1%, and HER2-negative (immunohistochemistry 0/1+ or negative by *in-situ* hybridization), metastatic or unresectable locally advanced disease, treatment with SG in the second or subsequent line for advanced disease, known germline *BRCA1/2* status (pathogenic/likely pathogenic vs. non-carrier) or documentation sufficient to ascertain it based on American College of Medical Genetics and Genomics (ACMG) and the Association for Molecular Pathology (AMP) guidelines and Human Genome Variation Society (HGVS) nomenclature [23,24], age ≥ 18 years.

Key exclusions were missing essential dates for time-to-event endpoints, unknown *BRCA1/2* status or the absence of outcome information precluding survival analyses.

Consecutive eligible patients were identified from institutional electronic medical records using a standardized case-report form. Because this was a retrospective, exploratory analysis, no *a priori* sample-size calculation was performed. Follow-up began at SG initiation and continued until death, last documented contact, or data cut-off (20 June 2025), whichever occurred first. Disease assessments and imaging using computed tomography of adequate regions were performed per local practice (typically ~12 weeks). Patients without an event were censored at the date of last valid assessment, and those lost to follow-up were censored at last contact.

### 2.4 Germline BRCA1/2 Assessment

Germline testing for *BRCA1/BRCA2* was performed locally in laboratories participating in external quality assessment using targeted Next Generation Sequencing (NGS) with confirmatory Sanger when required by local practice. Variant classification followed ACMG/AMP recommendations. For the primary analyses, pathogenic/likely pathogenic carriers were classified as g*BRCA1/2*m, while non-carriers formed the *BRCA*-wild-type (g*BRCA1/2*wt) group. Variants of uncertain significance were not considered gBRCA 1/2m in primary analyses. Due to the limited number of g*BRCA1/2*m cases, *BRCA1* and *BRCA2* were analyzed as a pooled g*BRCA1/2*m group (vs. g*BRCA1/2*wt), without gene-specific analyses. Although testing was performed across participating centers and local assays may have differed in panel composition/platform details, all laboratories applied accredited procedures and standardized variant interpretation frameworks.

### 2.5 Data Sources

Source data were collected from the initial breast cancer diagnosis to the last documented follow-up at each site. Data were collected using a standardized electronic case-report form using prespecified variable definitions and coding rules and were harmonized across sites before analysis. Collected variables included demographics, tumor characteristics, disease burden, prior systemic therapy, details regarding SG treatment and outcomes. Missing data were not imputed. Analyses were conducted on available cases, with denominators reported for each variable. Before statistical analysis, data underwent quality control procedures, including checks for completeness, range/plausibility, internal consistency and cross-variable concordance with site-level queries resolved where needed.

### 2.6 Study Objectives

The primary objective was to determine whether g*BRCA1/2* status is associated with clinical outcomes in patients with mTNBC treated with SG. Secondary objectives included comparisons of OS and PFS between g*BRCA1/2*m and g*BRCA1/2*wt subgroups.

Endpoints were defined as follows: PFS—time from SG initiation to documented progression (according to Response Evaluation Criteria for Solid Tumors [RECIST] v1.1 [25]) or death from any cause, whichever occurred first; OS—time from SG initiation to death from any cause.

### 2.7 Bias

To mitigate selection and information bias, we applied uniform eligibility criteria across centers—aligned with those of the ASCENT trial [14]—and enrolled consecutive SG-treated patients. We prespecified the exposure (g*BRCA1/2*) and outcomes (PFS, OS) with standardized criteria in imaging assessment (RECIST v1.1 [25]) and compared baseline characteristics between g*BRCA1/2*m and g*BRCA1/2*wt to assess group comparability. Survival models were further adjusted for prespecified clinical confounders (Eastern Cooperative Oncology Group Performance Status [ECOG] at SG initiation, age, presence of brain metastases, prior PARPi).

### 2.8 Statistics

Statistical analyses were conducted in Stata 19 (StataCorp LLC, College Station, TX, USA). The cohort size reflected all consecutive SG-treated patients accrued during the study period; no formal a priori sample-size calculation was undertaken. Categorical variables are presented as n (%) and compared using the χ^2^ test or Fisher’s exact test, as appropriate. Continuous variables are reported as medians (range) and compared with the Wilcoxon rank-sum test. PFS and OS were analysed using Kaplan–Meier curves, with medians and 95% confidence intervals (CIs) reported and groups compared by log-rank tests. Associations with outcomes were further examined using multivariable Cox proportional hazards models adjusted for prespecified clinically relevant confounders; additional candidate variables were considered if *p* < 0.20 in univariable analyses. To limit overfitting, the number of covariates entered into each model was constrained relative to the number of observed events. Proportional hazards assumptions were evaluated using Schoenfeld residuals. All tests were two-sided, with *p* < 0.05 indicating statistical significance.

## 3 Results

### 3.1 Baseline Characteristics

The median age at initiation of SG treatment was 53.0 years (interquartile range [IQR], 27.0–86.0). All patients were female. Most patients had recurrent disease (81.4%) and 80.7% had received chemotherapy in the early breast cancer setting. The predominant histopathological subtype was invasive carcinoma of no special type (NST) (91.7%). The most common sites of metastatic disease were lungs (53.8%), followed by liver (33.3%), bone (38.6%) and central nervous system (9.8%). Combined positive score (CPS) for PD-L1 was available in 91 patients (34.5%), of whom 40.7% had CPS ≥10. Details regarding baseline characteristics are presented in Table 1.

### 3.2 BRCA1/2 Status

Among the 264 patients with available g*BRCA1/2* results, 35 (13.3%) were g*BRCA1/2*m and 229 (86.7%) were g*BRCA1/2*wt. Patients with g*BRCA1/2*m were significantly younger than those with g*BRCA1/2*wt (*p* < 0.0001) and numerically had brain metastases more frequently. Other baseline characteristics were comparable between groups. A detailed comparison is provided in Table 1.

**Table 1:** Baseline clinical and pathological characteristics of patients with metastatic triple-negative breast cancer treated with sacituzumab govitecan, stratified by germline *BRCA1/2* status.

Characteristic		g*BRCA1/2* Mutated [n = 35]	g*BRCA1/2* Wild-Type [n = 229]	*p*-Value
Age, median (range)		43.9 (29.0–72.0)	54.7 (27.0–86.0)	<0.01*
Recurrent disease, n [%]		26 [74.3%]	189 [82.5%]	0.25
ECOG, n [%]	0	19 [54.3%]	93 [40.8%]	0.19
	1	14 [40.0%]	127 [55.5%]	
	≥2	2 [5.7%]	9 [3.9%]	
CPS tested, n [%]		9 [25.7%]	82 [35.8%]	0.34
CPS ≥10#, n [%]		3 [33.3%]	34 [41.5%]	0.73
Histopathological subtype, n [%]	NST	33 [94.3%]	209 [91.3%]	0.71
	Lobular	1 [2.9%]	4 [1.8%]	
	Metaplastic	0	8 [3.5%]	
	Other subtype	1 [2.9%]	8 [3.5%]	
Metastasis sites, n [%]	Lungs	21 [60.0%]	121 [52.8%]	0.47
	Liver	16 [45.7%]	72 [31.4%]	0.12
	Bones	15 [42.9%]	87 [38.0%]	0.58
	CNS	7 [20.0%]	19 [8.3%]	0.06
Number of prior lines before SG, n [%]	1	14 [40.0%]	129 [56.3%]	0.08
	2	12 [34.3%]	72 [31.4%]	
	3	6 [17.1%]	15 [6.6%]	
	≥4	3 [8.6%]	13 [5.7%]	
Prior therapy in MBC, n [%]	Pembrolizumab	1 [2.9%]	28 [12.2%]	0.14
	Anthracyclines	13 [37.1%]	88 [38.4%]	0.88
	Taxanes	13 [37.1%]	96 [41.9%]	0.71
	Carboplatin/Cisplatin	18 [51.4%]	123 [53.7%]	0.86
	Capecitabine	6 [17.1%]	56 [24.5%]	0.52
	PARP inhibitor	19 [54.3%]	0	<0.01*
	Gemcitabine	10 [28.6%]	69 [30.1%]	0.85
	Vinorelbine	4 [11.4%]	20 [8.7%]	0.54

Note: *, statistically significant; #, among tested n = 91; *gBRCA1/2*, germline *BRCA1/2*; SG, sacituzumab govitecan; ECOG, Eastern Cooperative Oncology Group (performance status); CPS, combined positive score; PD-L1, programmed death-ligand 1; NST, no special type; CNS, central nervous system; MBC, metastatic breast cancer; PARP inhibitor, poly(ADP-ribose) polymerase inhibitor; n, number.

### 3.3 Sacituzumab Govitecan Efficacy by Germline BRCA1/2 Status

**Figure 1:**
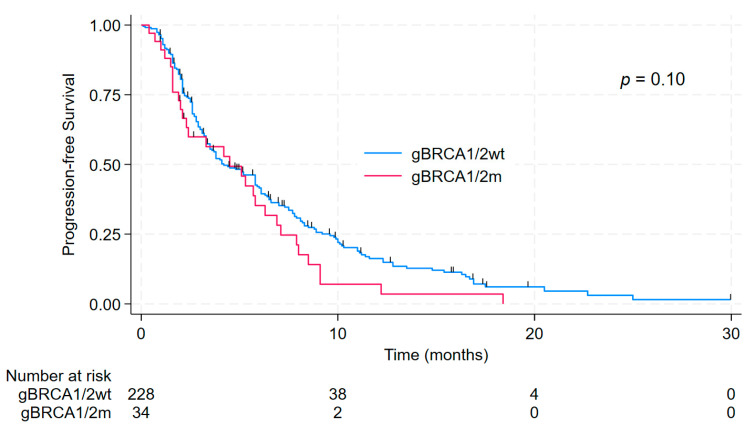
Kaplan–Meier curves for progression-free survival (PFS) according to germline *BRCA1/2* status. PFS is shown separately for patients with germline pathogenic variants in *BRCA1/2* (g*BRCA1/2*m) and those with germline wild-type *BRCA1/2* (g*BRCA1/2*wt), treated with sacituzumab govitecan.

After a median follow-up time of 9.9 months, the median PFS was 4.5 months (95% CI 2.1–6.3) in g*BRCA1/2*m compared to 4.2 months (95% CI 3.5–5.8) in g*BRCA1/2*wt patients (*p* = 0.10). Notably, the Kaplan–Meier survival curves crossed near the median, suggesting a trend toward earlier progression in g*BRCA1/2*m. At 6 months, PFS rates were 35.2% (95% CI, 18.9–52.0%) in g*BRCA1/2*m and 41.5% (95% CI, 34.8–48.1%) in g*BRCA1/2*wt patients. At 12 months, corresponding PFS rates were 7.0% (95% CI, 1.3–20.1%) and 16.2% (95% CI, 11.2–22.1%), respectively. These results are presented in Fig. 1. After adjustment for prespecified confounders in multivariable analysis, g*BRCA1/2* status was not significantly associated with PFS (Table 2). The proportional hazards assumption was evaluated using Schoenfeld residuals and showed no evidence of violation (*p* = 0.49).

**Figure 2:**
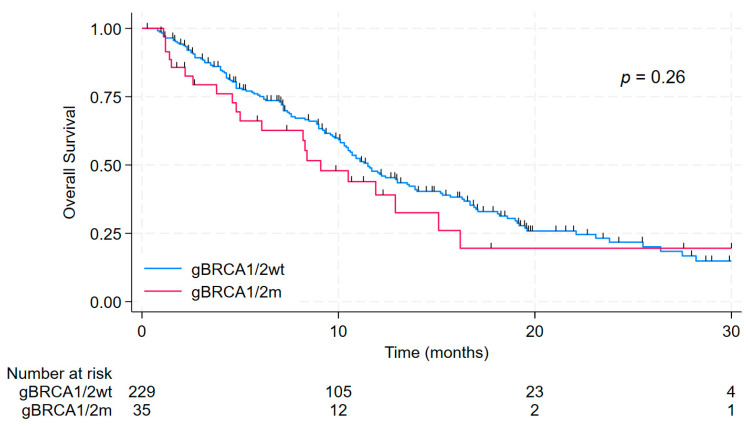
Kaplan–Meier curves for overall survival (OS) according to germline *BRCA1/2* status. OS is shown for patients with germline pathogenic *BRCA1/2* variants (g*BRCA1/2*m) versus germline *BRCA1/2* wild-type (g*BRCA1/2*wt), treated with sacituzumab govitecan.

The median OS was 9.1 months (95% CI 5.0–15.1) in g*BRCA1/2*m compared to 11.5 months (95% CI 10.3–13.5) in g*BRCA1/2*wt patients (*p* = 0.26). At 12 months, OS was 39.0% (95% CI, 21.1–56.7%) in g*BRCA1/2*m and 47.1% (95% CI, 39.8–54.1%) in g*BRCA1/2*wt patients. These results are presented in Fig. 2. In adjusted Cox models, g*BRCA1/2* status showed no independent association with OS. ECOG performance status was the sole independent predictor of poorer OS (Table 2).

Prior exposure to PARPi differed inherently between gBRCA1/2-mutated and wild-type patients. Among germline BRCA1/2 carriers (n = 35), 19 (54.3%) had previously received PARPi. In an exploratory subgroup analysis restricted to BRCA carriers, prior PARPi exposure did not impact PFS (log-rank *p* = 0.80). Similarly, in a univariable Cox model, prior PARPi use was not associated with PFS (HR 1.10; 95% CI 0.51–2.39; *p* = 0.80).

**Table 2:** Univariate and multivariate Cox regression analyses for progression-free survival and overall survival.

Progression-Free Survival				
Univariate Analysis				
Variable		HR	95% CI	*p*
g*BRCA1/2*	Yes vs. no	1.38	0.93–2.03	0.11
ECOG	≥1 vs. 0	1.13	0.86–1.48	0.37
Age	continuous	0.99	0.98–1.00	0.05
CNS mets	Yes vs. no	1.44	0.92–2.24	0.11
Prior PARPi	Yes vs. no	1.40	0.85–2.31	0.18
**Multivariate Analysis**				
g*BRCA1/2*	Yes vs. no	1.08	0.60–1.96	0.79
Age	continuous	0.99	0.98–1.00	0.13
CNS mets	Yes vs. no	1.44	0.92–2.65	0.12
Prior PARPi	Yes vs. no	1.14	0.55–2.38	0.72
**Overall Survival**				
**Univariate Analysis**				
**Variable**		**HR**	**95% CI**	***p***
g*BRCA1/2*	Yes vs. no	1.30	0.82–2.05	0.27
ECOG	≥1 vs. 0	1.90	1.38–2.63	<0.01*
Age	continuous	0.99	0.98–1.00	0.12
CNS mets	Yes vs. no	1.55	0.96–2.50	0.08
Prior PARPi	Yes vs. no	1.21	0.64–2.31	0.56
**Multivariate Analysis**				
ECOG	≥1 vs. 0	1.97	1.42–2.74	<0.01*
Age	continuous	0.99	0.98–0.99	0.05
CNS mets	Yes vs. no	1.50	0.93–2.43	0.10

Note: *, statistically significant; *gBRCA1/2*, germline *BRCA1/2*; ECOG, Eastern Cooperative Oncology Group (performance status); CNS, central nervous system; mets, metastases; PARP, poly(ADP-ribose) polymerase inhibitor; PARPi, PARP inhibitor.

## 4 Discussion

This is the first report to evaluate outcomes with SG by germline *BRCA1/2* status. We observed no differences in OS or PFS between g*BRCA1/2*m and g*BRCA1/2*wt patients. Carriers were significantly younger and showed numerically higher rates of brain metastases, with no cases of metaplastic breast carcinoma. Other baseline characteristics were broadly comparable. Findings were unchanged after adjustment for prespecified clinical confounders. Our findings complement a prior CEBCC analysis in SG-treated mTNBC focused on PD-L1 CPS testing patterns and outcomes, extending biomarker-oriented real-world evidence from this platform to germline BRCA1/2 status [21].

In other real-world studies and in the pivotal ASCENT trial [14], g*BRCA1/2* status was either not reported or presented only as a baseline characteristic, without subsequent analysis of treatment outcomes [15,16,17]. In our cohort, g*BRCA1/2* status was available for 87% of patients, reflecting a relatively high testing uptake compared to other real-world data, where up to 40% of patients had unknown status [26,27]. While historically, germline testing in metastatic breast cancer was pursued primarily for genetic counseling, its clinical implications have expanded substantially in recent years. With the approval of PARPi for patients with g*BRCA1/2*m, timely and comprehensive testing has become crucial in guiding therapy. Current guidelines [12,13] recommend olaparib in the adjuvant setting for high-risk HER2-negative patients as monotherapy or combined with endocrine therapy after (neo)adjuvant chemotherapy, based on the OlympiA trial. After a median of 6.1 years of follow-up, one year of olaparib in high-risk patients reduced the risk of invasive disease by 45% (HR 0.65, 95% CI 0.53–0.78), distant disease by 45% (HR 0.65, 95% CI 0.53–0.81) and reduced the risk of death by 28% (HR 0.72, 95% CI 0.56–0.93) [28,29]. In the metastatic setting, olaparib or talazoparib may be used as monotherapy for patients with g*BRCA1/2*m, HER2-negative tumors. Prior treatment with an anthracycline or/and taxanes, or with endocrine therapy for hormone receptor–positive disease, in the (neo)adjuvant or metastatic setting is expected unless these therapies are contraindicated or unsuitable [30,31]. In the OlympiAD trial, olaparib versus physician’s-choice chemotherapy reduced the risk of death when administered in the first line (HR 0.55, 95% CI 0.33–0.95) without statistical significance in the overall population (HR 0.89, 95% CI 0.67–1.18), and prolonged PFS (HR 0.58, 95% CI 0.43–0.80) with higher objective response rate (ORR, 59.9% vs. 28.8%) [32,33]. Similarly, in the phase 3 EMBRACA trial [34], talazoparib reduced the risk of disease progression (HR 0.54, 95% CI 0.41–0.71) with increased ORR (62.6% vs. 27.2%) and showed a non-significant reduction in the risk of death compared with chemotherapy (HR 0.85, 95% CI 0.670–1.073). The BROCADE phase 3 trial [35] in advanced breast cancer evaluated adding a PARPi to a platinum-based regimen, comparing paclitaxel–carboplatin with or without veliparib in g*BRCA1/2*m breast cancer. PFS favored the veliparib arm (HR 0.71, 95% CI 0.57–0.88), however, given the substantial hematologic toxicity of this combination, it is not recommended by current ABC guidelines [12].

It is essential to note that the pivotal, abovementioned PARPi trials enrolled patients who were largely naive to both ICIs and ADCs. Thus, the efficacy and biological activity of PARPi following prior exposure to pembrolizumab and/or SG have not been established. Conversely, it is also unclear how prior PARPi treatment may influence subsequent response to SG or other agents.

The optimal sequencing of pembrolizumab, PARPi, and SG in the treatment of mTNBC remains an open and clinically relevant question. g*BRCA1/2*m tended to receive SG later in the treatment sequence compared with g*BRCA1/2*wt patients, which is in line with current clinical guidelines. In current real-world practice, the typical sequence for patients with g*BRCA1/2*m has involved first-line pembrolizumab combined with chemotherapy, followed by PARPi in the second-line setting, and SG subsequently. However, this paradigm may shift with increasing consideration of SG in earlier lines of therapy, particularly in combination with pembrolizumab as per the ASCENT-04/Keynote-D19 clinical trial [36]. These gaps in evidence highlight the need for prospective trials or real-world data to inform sequencing strategies, particularly in g*BRCA1/2*m TNBC, where multiple active agents are available, but their optimal integration remains undefined. Early-phase efforts to combine PARP inhibition with SG are ongoing. In NCT04039230, evaluating SG with talazoparib, concurrent administration was limited by dose-limiting myelosuppression and poor tolerability (median PFS 2.3 months), whereas a sequential regimen (SG followed by talazoparib) was feasible, without dose-limiting toxicities, with a reported median PFS of 7.6 months and no new safety signals beyond the known SG profile [37]. In the phase II dose-expansion cohort of the same trial, a sequential schedule (SG on days 1 and 8 followed by talazoparib on days 15 to 21 with mandatory granulocyte colony-stimulating factor prophylaxis) showed preliminary activity, but hematologic toxicity remained substantial [38]. In the phase Ib SEASTAR (NCT03992131) arm B case series evaluating rucaparib plus SG, overlapping hematologic toxicity, particularly neutropenia, was dose-limiting and required dose modifications. Consequently, an optimal regimen and a recommended phase II dose were not established [39].

Another emerging clinical challenge is the optimal management of patients who received adjuvant olaparib and subsequently develop metastatic recurrence. In this context, the effectiveness of rechallenging with PARPi remains uncertain. Evidence extrapolated from ovarian cancer—where PARPi are the backbone of maintenance therapy—indicates that “PARP after PARP” provides only modest efficacy [40,41]. Accordingly, real-world evidence in breast cancer is needed to inform treatment decisions and refine sequencing strategies in this setting.

Interestingly, in our cohort, all cases of metaplastic breast carcinoma—a rare, aggressive histologic subtype [42] occurred exclusively among g*BRCA1/2*wt patients, suggesting potential phenotypic differences between g*BRCA1/2*m and g*BRCA1/2*wt mTNBC. This pattern contrasts with Corso et al. [43], who reported a higher frequency of metaplastic breast carcinoma in g*BRCA1/2*m (13/1114 = 1.2%) than in g*BRCA1/2*wt patients (10/4112 = 0.2%, *p* = 0.0002), corresponding to an increased risk of developing this histological subtype in carriers (OR = 4.47, 95% CI, 1.95–10.23). Similarly, Rodríguez-Fernández et al. [44] observed 3.2% metaplastic breast carcinoma among 93 g*BRCA1/2*m breast cancers versus 0.8% among 3157 g*BRCA1/2*wt.

This study has several limitations. Its retrospective, real-world design entails a risk of selection bias; inclusion was restricted to patients who received SG, limiting applicability to the broader mTNBC population and excluding g*BRCA1/2*m patients who, due to rapid progression or death, did not reach later lines—potentially underrepresenting the most aggressive cases. Because cohort entry occurred at SG initiation, earlier deteriorating patients were systematically excluded, which may have introduced immortal-time bias and slightly overestimated outcomes. The small number of g*BRCA1/2*m (n = 35) limits statistical power. Not all g*BRCA1/2*m patients received prior PARPi, which could have influenced subsequent outcomes and constrained inferences about optimal sequencing. Prior exposure to PARPi differs inherently between gBRCA1/2-mutated and wild-type patients, as only the former are standardly eligible for PARPi in metastatic breast cancer. This treatment history may introduce biological and clinical heterogeneity within the BRCA-mutated subgroup. However, in our exploratory stratified analysis restricted to gBRCA1/2 carriers, prior PARPi exposure was not associated with PFS, indicating that earlier PARPi treatment does not materially alter subsequent sensitivity to sacituzumab govitecan. These findings mitigate concerns that differences in prior PARPi use confound the association between BRCA status and outcomes with sacituzumab govitecan. Germline *BRCA1/2* testing and radiologic assessments were performed locally without central review, introducing potential variability in diagnostics and response evaluation, and residual confounding cannot be fully excluded despite multivariable adjustment. Furthermore, the relatively short median follow-up (9.9 months) limits the robustness of overall survival interpretation and constrains our ability to assess long-term survival differences. Finally, the cohort reflects practice patterns in Central/Eastern European CEBCC centers operating within specific testing and reimbursement frameworks, which may limit generalizability to other healthcare settings.

## 5 Conclusions

In this multicenter, international, real-world cohort of mTNBC treated with SG, germline *BRCA1/2* status was not associated with clinical outcomes. SG showed clinical activity irrespective of gBRCA1/2 status, including in patients previously exposed to PARPi. Although g*BRCA1/2*m patients had numerically earlier progression and a higher frequency of brain metastases, these observations require confirmation in larger, prospectively collected cohorts.

## Data Availability

The data that support the findings of this study are available from the Corresponding Author, [Mirosława Püsküllüoğlu], upon reasonable request.

## Supplementary Materials

The supplementary material is available online at https://www.techscience.com/doi/10.32604/or.2026.076384/s1.
